# In the literature: June 2020

**DOI:** 10.1136/esmoopen-2020-000832

**Published:** 2020-07-07

**Authors:** Desamparados Roda, Valentina Gambardella, Juan-Miguel Cejalvo, Andrés Cervantes

**Affiliations:** 1 Department of Medical Oncology, Biomedical Research Institute INCLIVA, CiberOn, University of Valencia, Valencia, Spain; 2 CIBERONC, Instituto de Salud Carlos III, Madrid, Comunidad de Madrid, Spain

**Keywords:** not applicable

## Single-cell analyses inform mechanisms of myeloid-targeted therapies in colon cancer

Immunotherapy based on checkpoint blockade has revolutionised cancer treatment during last years. Whereas this approach fails in a relevant group of patients, the knowledge on tumour microenvironment (TME) opened the possibility to the use of additional therapeutic strategies to potentiate antitumour immunity, including depletion of protumourigenic or immune suppressive and activation of specific immune populations using agonistic antibodies. Nevertheless, due to the complexity of the TME, many of these strategies have been indiscriminately advanced to the clinic without clear mechanistic hypotheses. Nowadays, single-cell RNA sequencing (scRNA-seq)-based transcriptome analyses identify T cell heterogeneity, elucidating dynamic relationships between T cells and the complexity of tumour-infiltrating myeloid cells, including tumour associated macrophages (TAMs) and dendritic cells (DCs), which contribute to malignancy through production of tumour and angiogenic growth factors, extracellular matrix remodelling and immunosuppression.^1^ Multiple strategies to boost the function of DCs and TAMs have advanced to the clinic, with approaches to activate the CD40 and CSF1R receptor being widely explored. However, CSF1R inhibitors and CD40 agonists have shown limited monotherapy efficacy.^2^

In a brilliant investigation recently published in Cancer Cell, Zhang *et al*, presented the results obtained by using two scRNA-seq platforms to perform a high-resolution analysis of immune and stromal cell populations in tumours, adjacent normal tissues and blood from colorectal cancer (CRC) patients.^3^ The authors were able to reproduce a cell–cell interaction network to define key cell populations involved in regulating tumourigenesis and antitumour immunity and identified specific populations of TAMs and DCs as central nodes of cellular interaction evaluating these characteristics in preclinical models and human cancers.

The authors demonstrated that two distinct TAM populations were present in CRC samples, consisting of C1QC+, involved in phagocytosis and antigen presentation, and SPP1+, implicated in angiogenesis. Neither of these populations fit the M1 and M2 dichotomous phenotypes. The presence of two TAMs populations in CRC confirms the theory that distinct characteristics of macrophages appeared to be dependent on their tissue/tumour origins. Therefore, TAMs heterogeneity, diverse function and cell–cell interaction, argues for developing more specific target strategies against CRC TAMs in the future. In fact, the authors described that SPP1+ TAMs population were consistently present in CRC samples, suggesting a key role in the CRC tumourigenesis. They identified murine TAM subsets that were both refractory to depletion by anti-CSF1R and showed similarity with human SPP1+ TAMs. Thus, the persistence of this SPP1+ population, and loss of proinflammatory C1QC + TAM populations, may represent a mechanism of resistance. In conclusion, together with the poor prognosis of patients with high SPP1+ and low C1QC+ TAM signatures, the specific depletion of SPP1+TAMs could ultimately lead to improved myeloid-targeted immunotherapy or enhanced combination with immune checkpoint blockade.

The importance of understanding DC subtypes was similarly demonstrated. First the authors confirmed the amplification of cDC1 population on antiCD40 treatment. The authors did confirm that CD40 agonist treatment significantly increased the frequency Ccl22 + cDC1 cells in mice models without drastic effects on other myeloid subsets. Importantly, signature genes of activated cDC1s positively correlated with favourable overall survival of CRC patients, suggesting anti-CD40 could have relevance in human cancer tumourigenesis. Second, anti-CD40 treatment increases effector memory CD8 + T cells and reduces exhausted CD8 + T population. Their human cell–cell interaction analyses predicted an unexpected interaction between the BATF3 + cDC1 population and both exhausted CD8 + T cells as well as a distinct population of the BHLHE40 + Th1 like cells. While no significant frequency changes in exhausted CD8 + T cells following anti-CD40 treatment was observed, the expression level of several inhibitory receptors such as PD-1 was reduced. Interestingly, a rapid expansion of Bhlhe40 + Th1 like CD4 + T cells, similar to those cells identified in Microsatellite Instable CRC,^4^ was observed on anti-CD40 treatment that preceded an increase in the frequency of memory CD8 + T cells. These Bhlhe40 + T cells may potentiate cDC1-mediated CD8 + T cell infiltration, expansion and antitumour function by providing positive feedback signals to cDC1 cells, in part via their high Cd40lg expression.

In conclusion, targeted cell-depletion studies will help further define the precise role of the intratumoural cellular interactions in CD40 agonist-mediated responses. This study provides an in-depth understanding of mechanisms regulating CD40 agonist activity and further supports their importance in immunotherapy. It also reveals previously unappreciated myeloid-T cell and myeloid-stromal connections within CRC, providing mechanistic insights for immunotherapies currently in clinical development, and demonstrates an approach for dissecting the role of specific tumour-associated immune populations through complementary single cell analysis.

## Targeting HER2 with trastuzumab deruxtecan in multiple advanced solid tumours

ERBB2 amplification and mutations have been recognised as drivers in tumour development and progression. The impacts of ERBB2 amplification on tumour growth have been detected in several solid tumours, in particular in breast cancer (BC) where the use of trastuzumab and other monoclonal antibodies, drug conjugated antibodies or tyrosine kinase inhibitors (TKi) have revolutionised the natural history of patients with advanced and locoregional disease. In gastric cancer, trastuzumab is so far the only drug able to improve clinical outcomes in patients with HER2 amplified advanced disease. Beyond breast and gastric cancer, there is a need to develop new treatment options for patients with ERBB2 amplified or ERBB2-mutant solid tumours, as HER2-expressing lung, bladder, colorectal and endometrial cancers.

ERBB2 mutations have been reported in many solid tumours, and recent clinical studies showed differential results with TKi. Neratinib achieved an overall response rate (ORR) of 12.5%–32% in ERBB2 mutant BC, whereas in similar patients with non-small cell lung cancer (NSCLC) had no or very limited benefit (0%–4% ORR). Furthermore, dacomitinib resulted in an ORR of 11.5% for ERBB2 mutant NSCLC, but no responses were observed among ERBB2 exon 20 insertion mutation, Y772dupYVMA, which is the most commonly observed mutation.^5^

A phase I trial tested trastuzumab deruxtecan (T-DXd), a humanised anti-HER2 antibody conjugated to a potent topoisomerase I inhibitor, in patients with advanced ERBB2-expressing or ERBB2-mutant solid tumours, demonstrating promising antitumour activity with an acceptable safety profile in patients with ERBB2-amplified breast or gastric cancer. In an interesting article, recently published in *Cancer Discovery*, Tsurutani *et al*, presented the outcome of the HER2-expressing non-breast/ non-gastric HER2-mutant solid tumour cohort.^6^ ERBB2 mutations in advanced lung adenocarcinomas are associated with worse prognosis compared with patients with other oncogene-driven advanced lung adenocarcinomas. In the ERBB2 amplified or ERBB2-mutant NSCLC subgroup, 55.6% (10/18) of patients had a confirmed ORR, with a median duration of response (DoR) of 10.7 months and median progression-free survival (PFS) of 11.3 months. In the ERBB2-expressing or HER2-mutant CRC subgroup, only 5% (1/20) of patients had a confirmed response, despite the fact that 35% of them presented a KRAS/NRAS mutation. Median PFS was 4.0 months. Among nine patients with HER2 immunohistochemistry 3+ CRC, the confirmed ORR was 11.1% (1/9) and the confirmed disease control rate was 100% (9/9). In addition to NSCLC and CRC, responses were also observed across a number of other tumour types, including HER2-expressing or HER2-amplified salivary gland cancer, biliary tract cancer, and endometrial cancer, and HER2-mutant non-amplified BC. In this group, the ORR was 27.3% (6/22) and the median DoR was not reached with a median PFS of 11.0 months. In conclusion, the 6.4 mg/kg recommended dose demonstrated encouraging preliminary antitumour activity with an acceptable safety profile in patients with heavily pretreated ERBB2-amplified and/or ERRB-mutant solid tumours. Although sample sizes are too small to draw firm conclusions relative to specific tumour types, T-DXd showed promising antitumour activity in patients with ERBB2-mutant, especially in NSCLC. Effective anti-HER2 agents such as T-DXd may make the treatment of ERRB2 mutant tumours an agnostic indication.

## Alterations in *PTEN* and *ESR1* promote clinical resistance to alpelisib plus aromatase inhibitors in PI3K-mutant BCs

Around 75% of BCs are hormone receptor-positive (HR+) and HER2-negative. Among them, 40% have activating mutations in the *PIK3CA* gene. *PIK3CA*-mutated cancers have been shown to be sensitive to PI3K inhibitors. Alpelisib, an α-specific PI3K inhibitor, has been developed and approved for clinical use in combination with hormonotherapy. The SOLAR-1 phase III trial of alpelisib plus fulvestrant in *PIK3CA*-mutated HR+ metastatic BC showed an improved PFS over fulvestrant monotherapy.^7^ However, despite this relevant clinical benefit, resistance remains a challenge.

Razavi *et al* published in Nature Cancer an inspiring article that performed a longitudinal analysis of tumour and plasma circulating circulating-tumour DNA (ctDNA) among patients with *PIK3CA*-mutant HR + metastatic BC, who participated in a phase I/II dose-escalation study of alpelisib in combination with aromatase inhibitors (letrozole or exemestane).^8^ The most frequent grade 3 adverse event of was a dose-dependent maculopapular rash. As we expected, clinical benefit was only observed in patients whose tumours harboured *PIK3CA* mutations. The purpose of this research was to identify potential mechanism of resistance. The authors analysed a combination of pre-treatment tumour biopsies and ctDNA samples as well as post-treatment ctDNA samples from participants in the trial. First, they described that the majority of *PIK3CA* mutations (70%) were clonal. Second, they assessed whether any co-occurring alterations were associated with clinical benefit. The presence of *ESR1* mutations in baseline tumours was significantly associated with lack of clinical benefit. Furthermore, they identified two patients with concurrent *PIK3CA* mutations and *PTEN* loss, who presented early progressive disease without clinical benefit. Interestingly, to address the spatial and temporal distribution of genomic alterations observed in advanced BC, the authors used preprogression and postprogression liquid biopsy. They detected *PTEN* mutations in 25% of patients, including three patients with loss-of function alteration in pretreatment sample (who had rapid progression of disease), and five patients with emerging mutations in post-treatment ctDNA. Also, they observed a significant increase in *ESR1* mutations between post-treatment and pretreatment ctDNA samples. To confirm if *ESR1* mutation might indeed contribute to tumour progression in these cases, they used CRISPR-based *ESR1* knock-out MCF7 cells. In vivo xenograft model revealed that *ESR1*-mutant tumours progressed faster to alpelisib plus oestrogen deprivation than parental tumours.

Beyond the association between *PTEN* loss, *ESR1* mutations and treatment resistance, this work finally explored whether other genomic alterations were acquired under selective pressure of therapy. This analysis revealed expansions of multiple alterations involving genes related with PI3K and MAPK pathway, taking into account the important cross-talk between PI3K and ER signalling in BC. Overall, the manuscript suggests that resistance to alpelisib in combination with aromatase inhibition might be mediated through *PTEN* and *ESR1* mutations. In fact, patients with these specific alterations might be considered for trials of oral selective ER degraders and/or AKT inhibitors.
